# Development of a Multiple-Locus Variable number of tandem repeat Analysis (MLVA) for *Leptospira interrogans *and its application to *Leptospira interrogans *serovar Australis isolates from Far North Queensland, Australia

**DOI:** 10.1186/1476-0711-4-10

**Published:** 2005-06-30

**Authors:** Andrew T Slack, Michael F Dohnt, Meegan L Symonds, Lee D Smythe

**Affiliations:** 1 WHO/FAO/OIE Collaborating Centre for Reference & Research on Leptospirosis, Centre for Public Health Sciences, Queensland Health Scientific Services, Brisbane, Australia

## Abstract

**Background:**

Leptospirosis is a zoonotic disease caused by the genus, Leptospira. *Leptospira interrogans *is the most common genomospecies implicated in the disease. Epidemiological investigations are needed to distinguish outbreak situations or to trace reservoirs of the organisms. Current methodologies used for typing Leptospira have significant drawbacks. The development of an easy to perform yet high resolution method is needed for this organism.

**Methods:**

In this study we have searched the available genomic sequence of *L. interrogans *serovar Copenhageni strain Fiocruz L1-130 for the presence of tandem repeats [1]. These repeats were evaluated against reference strains for diversity. Six loci were selected to create a Multiple Locus Variable Number of Tandem Repeats (VNTR) Analysis (MLVA) to explore the genetic diversity within *L. interrogans *serovar Australis clinical isolates from Far North Queensland.

**Results:**

The 39 reference strains used for the development of the method displayed 39 distinct patterns. Diversity Indexes for the loci varied between 0.80 and 0.93 and the number of repeat units at each locus varied between less than one to 52 repeats. When the MLVA was applied to serovar Australis isolates three large clusters were distinguishable, each comprising various hosts including *Rattus *species, human and canines.

**Conclusion:**

The MLVA described in this report, was easy to perform, analyse and was reproducible. The loci selected had high diversity allowing discrimination between serovars and also between strains within a serovar. This method provides a starting point on which improvements to the method and comparisons to other techniques can be made.

## Background

Leptospirosis, is the zoonotic disease caused by the spirochete Leptospira. Leptospirosis is characterised either as a febrile illness with sudden onset, or a 'flu like' illness. Patients present with chills, headaches, myalgia, and abdominal pain. In addition, patients may present with renal and pulmonary complications. It is considered an emerging infectious disease with large documented outbreaks occurring world-wide [2]. The majority of isolates detected in Queensland, Australia belong to the genomospecies, *L. interrogans*, with the dominant serovars being Zanoni or Australis. Typically Leptospirosis infections in Queensland are a result of occupational exposure with the majority of cases occurring in farming or animal based industries [3].

The genus Leptospira contains 17 genomospecies as shown by DNA-DNA hybridisation [4]. Under the current genotypic classification system, pathogenic and non pathogenic serovars may reside within the same genomospecies [2]. Many animals both domesticated and native serve as the reservoir for this bacterium. Leptospires are shed into the environment via the urine of these animals, where they survive in the soil or freshwater. Human infections result from contact with the contaminated soil/water or from direct contact with animals or their infectious body fluids [5].

Epidemiological investigation of this organism is important for the ability to distinguish individual cases from related cases such as in an outbreak situation. Also of importance is determining which animal is the likely common source of the infection, as containing or preventing the spread of the vector is paramount to controlling the disease.

Molecular typing methods have been described for *L. interrogans *including Randomly Amplified Polymorphic DNA (RAPD) [6-9], Pulsed Field Gel Electrophoresis (PFGE) [10], Arbitrarily Primed PCR (AP-PCR) [11,12] and most recently Fluorescent Amplified Fragment Length Polymorphism (FAFLP) [13]. Each of these methods have their disadvantages such as insufficient discriminatory power, poor inter-lab and intra-lab reproducibility, difficulties with database and dissemination of data [14]. They may also require specialised equipment such as DNA sequencers or contour-clamped homogeneous electric field electrophoresis systems. In addition leptospires have their own particular problems when using the above methods, the fastidious nature of the organism does not easily allow for the large volume of culture material required for PFGE and cultures can be quite prone to contamination with other bacteria, which may influence the accuracy of low stringency PCR methods or AFLP. As an alternative to the above methods, investigation of Variable Number of Tandem Repeats (VNTR) has been described for various organisms. These include *Salmonella enterica *[14,15], *Staphylococcus aureus *[16], *Yersinia pestis *[17], *Mycobacterium tuberculosis *[18], *Francisella tularensis *[19], *Legionella pneumophila *[20], *Brucella spp*. [6,21], *Escherichia coli O157:H7 *[22]and *Borrelia spp*[23]. VNTR are repeated DNA sequences of varying copy number. They are caused by slipped strand mispairing during DNA replication [24,25]. VNTRs can provide information relating to both the evolutionary and functional areas of bacterial diversity[25]. The ability to detect VNTRs in micro-organisms has been greatly enhanced by the availability of whole genomic sequences and software that can search for VNTR loci from these sequences. 1, [26,27] Once these polymorphisms are located, flanking primers can then be designed to amplify these variable length regions thus allowing differentiation of copy numbers using the size of the resultant amplicon. This can be done using standard agarose gel electrophoresis and if a higher resolution is required, fluorescent labelling and fragment sizing via a DNA sequencer can be used. VNTR is therefore applicable to a wide range of laboratories, including those which may have simple equipment such as thermal cyclers and agarose gel electrophoresis but do not have access to sophisticated equipment such as DNA sequencers. Furthermore when VNTR is applied to multiple loci as a typing scheme such as in Multiple Locus VNTR Analysis (MLVA) greater discriminatory power and more accurate determination of genetic relatedness is achieved [17,19,28,29]. Recently Majed et al [30]described a MLVA typing scheme for *L. interrogans *Sensu Stricto, this research highlighted the value of using VNTR as a typing scheme but was limited to the identification of serovar by comparing the size of the repeat to that of known reference strains. It should be noted that in that study several reference strains had identical VNTR patterns including serovars Australis and Bratislava, serovars Copenhageni and Icterohaemorragiae and serovars Romanica and Wolffi. It was subsequently validated against a small number of clinical isolates. In this paper, we report on the development of a MLVA scheme using novel VNTR loci selected from the sequence of a published *L. interrogans *genome [1] and evaluate its usefulness as a phylogenetic typing method using reference strains and clinical isolates from Far North Queensland, Australia.

## Methods

### Bacterial Strains

Thirty-nine reference strains of *L. interrogans *were obtained from the reference culture collection maintained by the WHO/FAO/OIE Collaborating Centre for Reference & Research on Leptospirosis, Brisbane, Australia (Table 1). In addition to the reference strains, ninety-eight isolates of *L. interrogans *serovar Australis were analysed [Additional File One]. These isolates were recovered from human and animal specimens. Human isolates were cultured using 0.2–0.5 mL of whole blood inoculated into 3 mL of semi solid Ellinghausen McCollugh Johnson Harris broth supplemented with 0.15% agar (EMJH, Difco lab, USA). These were then sub-cultured into EMJH broth within one week of receipt at the laboratory. Cultures were incubated for a further six weeks at 30°C and inspected weekly using dark ground microscopy. Positive cultures were identified using hyperimmune antisera and the Cross Agglutination Absorption Test (CAAT). 3 mm cubes of kidney or 100 μL of urine from rodents were inoculated into 3 mL semi solid EMJH media, these were incubated at 30°C for six weeks and inspected weekly using dark field microscopy. Positive cultures were identified as above. Once identified, isolates were stored in liquid nitrogen using EMJH media containing 2.5% dimethyl sulfoxide (DMSO).

**Table 1:** Leptospira interrogans reference strains used for VNTR loci selection.

**Serogroup**	**Serovar**	**Strain**	**Area of isolation**	**Source**
Australis	Australis	Ballico	Australia	Human
Pomona	Pomona	Pomona	Australia	Human
Sejroe	Medanesis	Hond HC	Indonesia	Dog
Icterohaemorrhagie	Copenhageni	M20	Denmark	Human
Mini	Swaijak	Swaijak	Australia	Human
Sejroe	Hardjo	Hardoprajitno	Indonesia	Human
Icterohaemorrhagie	Icterohaemorrhagie	Ictero 1	Japan	Human
Autumnalis	Autumnalis	Akiyami A	Japan	Human
Canicola	Canicola	Hond Utrecht IV	Netherlands	Dog
Australis	Muenchen	Munchen C90	Germany	Human
Australis	Fugis	Fudge	Malaysia	Human
Australis	Lora	Lora	Italy	Human
Autumnalis	Weerasinghe	Weerasinghe	Sri Lanka	Human
Bataviae	Bataviae	Swart	-	-
Bataviae	Paidjan	Paidjan	Indonesia	Human
Canicola	Benjamini	Benjamin	Indonesia	Human
Canicola	Binjei	Binjei	Indonesia	Human
Canicola	Broomi	Patane	Australia	Human
Djasiman	Djasiman	Djasiman	-	-
Icterohaemorrhagie	Gem	Simon	Sri Lanka	Human
Pyrogenes	Abramis	Abraham	Malaysia	Human
Pyrogenes	Biggis	Biggs	Malaysia	Human
Pyrogenes	Camlo	Lt64-67	Vietnam	Human
Sejroe	Geyaweera	Geyaweera	Sri Lanka	Human
Pyrogenes	Zanoni	Zanoni	Australia	Human
Pyrogenes	Robinsoni	Robinson	Australia	Human
Autumnalis	Bangkinang	Bangkinang 1	Indonesia	Human
Autumnalis	Carlos	C3	Phillippines	Toad
Autumnalis	Mooris	Moores	Malaysia	Human
Bataviae	Losbanos	LT101-69	Phillippines	Rat
Canicola	Sumneri	Sumner	Malaysia	Human
Canicola	Jonsis	Jones	Malaysia	Human
Djasman	Sentot	Sentot	Indonesia	Human
Djasiman	Gurungi	Gurung	Malaysia	Human
Pyrogenes	Guaratuba	An7705	Brazil	Opossum
Icterohaemorrhagie	Smithi	Smith	Malaysia	Human
Sejroe	Wolffi	3705	Indonesia	Human
Sejroe	Ricardi	Richardson	Malaysia	Human
Sejroe	Haemolytica	Marsh	Malaysia	Human

### DNA Extractions

Once the cultures had reached a density equivalent to a 0.5 McFarland standard (1.5 × 10^8 ^cells/mL), cells were harvested by aspirating 500 μL of culture, centrifuged at 12,000 g for 4 minutes and resuspended in 200 μL of Phosphate Buffered Saline (PBS). DNA was extracted using the Roche High Pure Template kit as per manufacturer's instruction.

### VNTR primer design

The two chromosomes of *L. interrogans *Copenhageni strain Fiocruz L1-130 deposited in GenBank under accession numbers, NC005823 and NC005824 were used to detect the VNTR Loci. Analysis using the Tandem Repeat Finder (TRF) program [1,26] was used to identify potential VNTR loci. Primer Premier 5.0 (Premier Biosoft) was used to design PCR primers for amplifying the loci. Primers were designed within the flanking regions, with a theoretical melting temperature of 57°C to 60°C (Table 2).

**Table 2:** PCR primers used in Study

**Primer Name**	**Direction**	**Sequence (5'-3')**	**Theoretical Melting Temperature (°C)**
V8	Forward	CAA GTG TTC GAC AAG AAT GAG	57.4
	Reverse	CTC ACC GGT AGA ACG CTC TTT T	58.4
V27	Forward	TCG TCG GGT GAG CTA AAA TAT	57.0
	Reverse	TTC TTT CGG TGG CAA GG TTT	59.8
V29	Forward	ATC GTT TTG GCA GTT TTT GCT	57.7
	Reverse	CTA GAA AAT TCC GCG TAG GG	57.2
V30	Forward	AAG TAA GAT AGG TTC GGC GTT TA	57.9
	Reverse	ACT TGG GTG TTA ATC GCA AAA	57.7
V36	Forward	TGG TTC TTG GGG TAA TTC TGT T	58.2
	Reverse	CTA CCA GGA GAT TAT CAA AAC GAA	57.9
V50	Forward	CTT GTT GGA TCA CAA TAC GAA CTA TA	58.4
	Reverse	GGTAAGGGACAAAGTAAGTGAAGC	58.9

### VNTR PCR amplification

PCR amplification of the VNTR loci was performed in a total volume of 50 μL containing 1X PCR Buffer II (Applied Biosystems, Foster City, Calif.), 2 mM MgCl_2_, 200 μM dNTP mix (Amersham Pharmacia Biotech, Piscataway, N.J), 10 pmol each of forward and reverse primer (Table 2), 1 unit of Amplitaq Gold (Applied Biosystems, Foster City, Calif.), 2 μL of the DNA preparation and double distilled water (ddH_2_O) making up the volume to 50 μL. The PCRs were run on a GeneAmp 9700 thermal cycler (Applied Biosystems, Foster City, Calif.). An initial denaturation at 95°C for 9 minutes, was followed by 35 cycles of a three step cycle protocol: 94°C for 30 seconds, 58°C for 60 seconds and 72°C for 60 seconds and a final extension of 72°C for 7 minutes. Each PCR product (15 μL) was resolved by electrophoresis (2 hours at 80 V) through a 2% agarose gel containing 0.5 ug ethidium bromide and buffered with 1X TBE (90 mM Tris-borate, 1 mM EDTA, pH 8). Allelic sizes were estimated using a 100 bp DNA plus Ladder (MBI Fermentas, Vilnius, Lithuania) as a size marker. Gels were visualised using UV transillumination and the images captured using the ChemiDoc XRS System (BioRad, Hercules, Calif.) (Figure 2).

### Sequencing

The PCR products from eight selected references strains were sequenced using the same primers used to amplify the products. PCR product clean-up was performed using an enzyme digestion containing 1 μl of 10X Antarctic Phosphatase buffer (New England Biolabs, Beverly, Mass), 2 units of Exonuclease I (MBI Fermentas, Vilnius, Lithuania), 2 units of Antarctic Phosphatase (New England Biolabs, Beverly, Mass), 7 μL of PCR product and ddH_2_O to the final volume of 10 μL. This mix was incubated for 20 min at 37°C follow by 5 min at 80°C to inactivate the enzymes. Sequencing was performed using 1 μL of Big Dye Terminator V3.1 ready reaction mix (Applied Biosystems, Foster City, Calif.) with 7 μL of 2.5x Applied Biosystem sequencing dilution buffer, 3.2 pmol of primer, 3 μL of PCR product and ddH_2_O to the final volume of 20 μL. The thermal cycling was perform according to the manufacturer's instructions with the exception of increasing the cycles to 45. The sequencing products were cleaned up using sodium acetate-ethanol precipitation before being run on an ABI-373 sequencer. The sequences were aligned and analysed using Vector NTI Suite 9 (Invitrogen, Carlsbad, Calif.).

### Data Analysis

Using the Quantity One 1D Analysis software package (BioRad, Hercules, Calif.), the agarose gel images were analysed and allelic sizes estimated. Allelic sizes were then converted into repeat copy numbers using Microsoft Excel software package [Additional File One], using the formula: Number of Repeats (bp) = [Fragment size (bp) – Flanking regions (bp)] / Repeat size (bp). The repeat copy numbers were then rounded down to form whole numbers. When repeat numbers were less than one, they were rounded down to zero, whilst no amplification was represented by the number ninety-nine. This created a numerical profile which was analysed as a character dataset using Bionumerics software package version 3.5 (Applied-Maths, Sint-Martens-Latern, Belgium). Clustering analysis was done using the categorical parameter and the Ward coefficient. Nei's Diversity Index of the VNTR loci was calculated from the range of alleles generated from the reference strains utilising the formula; D = 1-Σ(allele frequency)^2 ^[31].

## Results

### Identification of VNTR markers

The Tandem Repeat Finder program identified 189 repeat motifs within the genome of *L. interrogans *serovar Copenhageni strain Fiocruz L1-130. 186 of the repeats were identified from chromosome 1 and only three were found in chromosome 2. 53 repeats were identified as being suitable for further analysis based upon the size of the repeat, number of repeat units present and also whether the sequence was conserved within the repeats. Preliminary testing against the reference strains identified 25 loci out of the 53 that were polymorphic between different serovars. The remaining 25 loci either failed to amplify any DNA or were amplified but were monomorphic. A subset of the *L. interrogans *serovar Australis clinical isolates that were considered geographically unrelated was used to determine whether the 25 selected loci were also polymorphic within a serovar. Six loci were found to contain variable repeat copy numbers within a serovar and were then re-applied to the 39 reference strains. Amplification of the six loci was possible from the 39 reference strains tested with the exceptions of locus V8 from serovar Djasiman, locus V27 from serovar Swaijak and Robinsoni, locus V29 from serovars Swaijak, Canicola, Broomi, Robinsoni and Jonsis. Amplification was also not possible for locus V36 from serovars Munchen and Fugis also for Locus V50 in serovars Lora and Geyaweera. PCR amplification was attempted three times for these serovars, no amplicons were detected at each attempt. The different allele sizes were caused by the loss or addition of repeat units confirmed by the sequencing of the PCR products. Sequence data was entered into GenBank [GenBank: DQ023538 – DQ023553].

For the 39 reference strains the number of repeats in the six loci varied between no repeats in all loci up to 52 repeats in the V36 locus. The number of alleles per locus varied between six in V27 and nineteen in V36. The diversity index ranged from the lowest of 0.80 in V27 to 0.93 in V36. (Table 3)

**Table 3:** Characteristic of the Six VNTR loci

Loci	Repeat Motif	Repeat size (bp)^a^	Total Flanking Regions (bp)^a^	Repeat range (min-max)	No. of alleles	Diversity (D)
8	GGAAAACTCAACACAA CGCTCTTTATGAATCG CGTT	36	124	0–16	14	0.88
27	TTGTGGGAACTCTTAC AATTTGAGATTTTACA GTAAAACTTGGAAGTT GTGGGAACTCTTACAA TTTGAGATTTTACAGTA AAACTTGGAAATTGTG GGAACTCTTACAACTT GAGATTTTACAGTGGG ACTTTGAAG	138	183	0–4	6	0.80
29	GATTTTACAGTTAGAC TTTGAAATTGTGGGAA CTCCCACGGATTTGG	47	90	0–17	14	0.92
30	TCCCACATATTCAAGA TTAAACTGTAAAATTGT GATTTGTGGTAGT	46	228	0–12	12	0.88
36	CTTAGACTTTGTGTGA GTTCCCACATTTTAAA GTAAAA	38	161	0–52	19	0.93
50	AAAATGTAGGAACTAC CACAAACACTGACTTT ACAGATAAATTCTC	46	106	012	9	0.83

### *L. interrogans *reference strains clustering analysis

**Figure 1:**
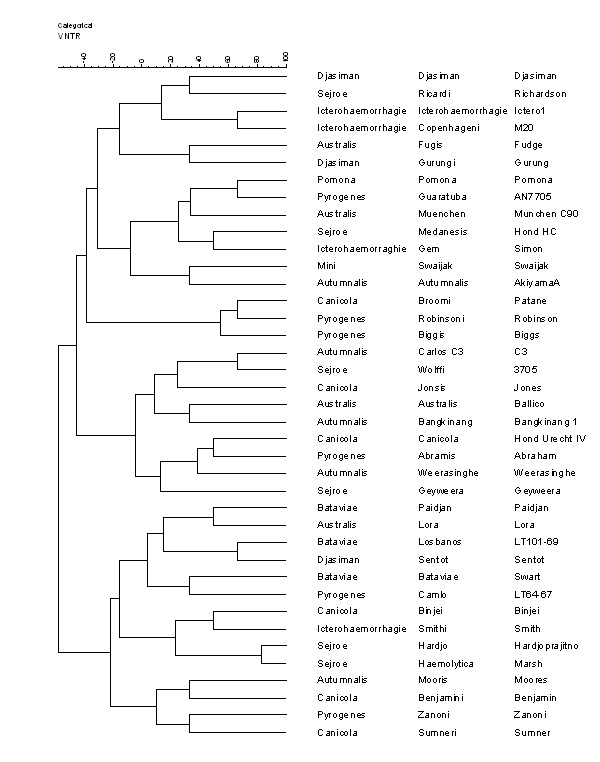
**Leptospira interrogans reference strains: clustering analysis using MLVA Data. **Clustering analysis was done using the categorical and ward options using Bionumerics software package version 3.5 (Applied-Maths, Sint-Martens-Latern, Belgium).

Clustering analysis (Figure 1) positioned the reference strains into three large clusters. These clusters each contained a diverse selection of serovars with no bias towards the grouping of serogroups together, with the exception of serovar Iceterohaemorrhagie strain Ictero 1, Copenhageni strain M20, serovar Hardjo strain Hardjoprajitno and Haemolytica strain Marsh. Both of these pairs of serovars belong to the same serogroups: Icterohaemorrhagie and Sejroe respectively.

### *L. interrogans *serovar Australis clustering analysis

To further evaluate the VNTR loci selected for the MLVA, the typing scheme was applied to 98 isolates of *L. interrogans *serovar Australis. All six VNTR loci were amplified from all of these clinical isolates, which varied in geography of isolation and host. Clustering analysis [Additional File Two and Additional File Three] revealed three major clusters, each containing several sub-groups. Three clusters contained a mix of Rattus species and human whilst two contained canine isolates. Two of the major clusters show significant geographical distributions towards the two main townships in the area: Tully and Innisfail, whilst the remaining major cluster was more diverse in geographical distributions. As the isolates were taken over a limited timeframe, not surprisingly there was no discernable pattern in regards to the introduction or extinction of strains over time in the area.

## Discussion

The MLVA assay presented was easy to perform and analyse, as it consisted of six individual PCR reactions and agarose gel electrophoresis. Selected reference strain isolates were run in tandem during the initial evaluation by different individuals to assess reproducibility, each time they displayed identical fragment sizes as determined by sequencing and agarose gel electrophoresis (data not shown). Dilutions of the template DNA were used to evaluate whether the fragment sizes were template concentration dependant. Dilutions of 1:10 and 1:100 did not effect fragment size but did result in reduced PCR product yield (data not shown). The MLVA assay was proven to be reproducible under varying laboratory conditions. The two limitations of this assay are firstly the use of agarose gel electrophoresis to separate fragments for allelic sizing, due to inherent inaccuracies of this method to size bands of close molecular weights given that the resolution is dependant on agarose composition and concentration and secondly in rounding partial repeat copy numbers to the nearest whole number to make the data analysis easier, isolates that had partial repeats were treated as if they contained whole repeats. As illustrated in Additional file 1, by simplifying the repeat copy numbers we have artificially reduced the resolution of the method and its ability to distinguish between closely related strains that may only vary by a partial repeat at one or more of the loci.

The diversity index calculated for each locus suggests that the loci selected are of highly polymorphic nature and therefore have greater discriminatory power between similar strains than loci with a lower diversity indexes would have. Whilst it has been previously reported in organisms such as *Francisella tularensis *[19] and *Yersinia pestis *[29] that higher copy repeat numbers may confer higher allelic variability, it was not demonstrated with this study. This may be due to the loci having similar repeat copy sizes (36–47 bp) with the exception of locus V27 with a repeat size of 138 bp. The lack of amplification from loci V8, V27, V29, V36 and V50 from certain reference strains may be due to sequence diversity in the flanking regions up or downstream from the repeat regions or the lack of the VNTR loci all together. Further investigations using isolates of these serovars is needed to determine whether there is sufficient diversity in the remaining loci for it to be valid as a typing method. In the study by Majed et al. [30], they noted from the dendrogram that the isolates had clustered into distinct global geographical regions. Interestingly in this study, we found that the dendrogram showed no bias towards this global geographical clustering of reference strains.

The different genomospecies of *Leptospira *were not used in the selection and development of the VNTR loci for this typing scheme. The basis for this decision was that the other genomospecies are considered to be significantly different from *L. interrogans *based upon DNA-DNA Hybridisation [32,33] and may not possess the same primer binding sites or indeed the same VNTR loci. In addition due to the use of a serologically based Leptospiral taxonomy system, several serovars belong to more than one genomospecies [32,33], thus it would be doubtful that the MLVA typing scheme described in this article would be useful in determining the genomospecies of these related serovars due to the significant genetic differences[34]

When the MLVA assay was applied to *L. interrogans *serovar Australis isolates collected from 1995 to 2004, the six selected loci appeared to show less diversity. This could be due to the fact that the isolates were taken from within a limited geographical area of Far North Queensland. Despite this apparent limited diversity, the phylogenetic analysis revealed several large albeit weakly linked clusters. All of these clusters contained a mixture of hosts, and it would be possible to speculate that the transmission of serovar Australis to humans in that area is via the native rodent population indigenous to that area. Another possible risk whilst not proven in Australia, is that transmission of the organism may occur via canines to humans [2]. Indeed in this study the two strains isolated from canines are genetically similar to two strains isolated from humans. Also of interest, the reference culture for serovar Australis; strain Ballico which was isolated from a patient in North Queensland in 1934, shows homology with LT958 and QHR371A a human and *Rattus sordidus *isolate respectively, both from the Tully region. Further investigations using MLVA of isolates, could add further detail to the depth of knowledge into the population of serovar Australis and to determine other possible transmission sources to humans.

Further improvements to this method are possible to increase both practicality and discriminatory power for typing of *L. interrogans *isolates. These improvements could include the use of fluorescently labelled primers and fragment analysis using a DNA sequencer to accurately assign repeat sizes. Multiplexing of the six targets would rationalise the number of PCR reactions needed to complete the MLVA and would also decrease costs in terms of reagents and labour. Improvements that could be introduced to the analysis of the data may include using an allele designation system as described by Lindstedt et al [34]. In addition to improving the method, comparisons between other molecular typing methods such as FAFLP or a sequence based typing scheme, would ultimately determine the validity of the MLVA assay as molecular epidemiology tool for *L. interrogans*.

The method has potential application in furthering the understanding of Leptospiral molecular epidemiology. As this method can be performed without specialised equipment, a broader range of laboratories including those in developing countries could potentially use this scheme as part of their isolate typing. This method was also easily standardised within our laboratory, with multiple users and different thermal cyclers employed to achieve the same results. This level of standisation at an inter laboratory level would allow the transfer of the method into another laboratory more effectively than that of a method that was operator or equipment specific. The simplification of MLVA data into a concise and portable numerical format as suggested in this article makes it easier to be comprehended by non-technical staff such as public health authorities. In addition, the format of allele data is similar to the allele string that is used for Multiple Locus Sequence Typing (MLST). As an alternative to using Bionumerics, software freely available on the internet such as Sequence Type Analysis and Recombinational Tests (START) [35] could be used for the phylogenetic analysis. Bionumerics was ultimately selected over START due to the advanced features of Bionumerics including evolutionary and population modelling.

Further assessment of *L. interrogans *isolates globally is required to confirm that the selected VNTR loci possess sufficient diversity to be used a typing scheme on an international level.

## Conclusion

We have developed a novel MLVA typing scheme which is simple, robust reproducible and cost effective. The six VNTR loci chosen for this assay showed a high level of diversity between reference strains. When this method was applied to a collection of clinical isolates, it was possible to observe distant relationships between suspected reservoirs and humans. This method provides a starting point for further investigations into the molecular epidemiology of *L. interrogans *infections.

## Competing interests

The author(s) declare that they have no competing interests

## Authors' contributions

AS had primary responsibility for study design, conducting the typing work and preparation of the manuscript. MD provided laboratory support by culturing and maintaining culture collections. MS provided laboratory support by performing serological identification of isolates. LS had intellectual contributions. All authors have read and approved the final manuscript.

**Figure 2:**
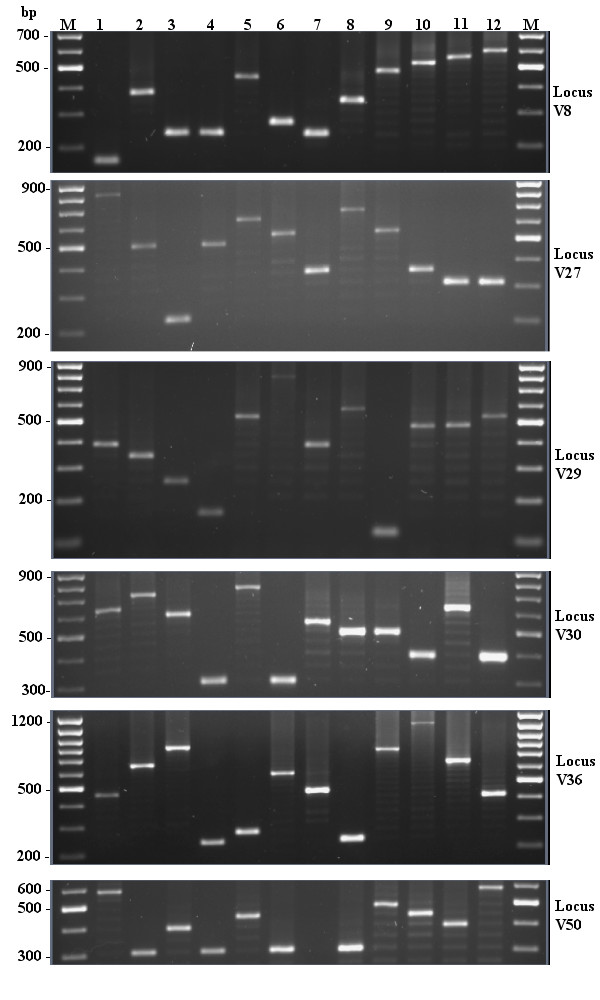
**PCR products from the six selected VNTR loci of various *L. interrogans *reference strains. **PCR products electrophoresised through a 2% agarose gel. M: 100 bp DNA Ladder plus (MBI Fermentas, Vilnius, Lithuania); 1, Serovar Zanoni strain Zanoni; 2, Serovar Autmnalis strain Akiyami A; 3, Serovar Canicola strain Hond Utrecht IV; 4, Serovar Pomona strain Pomona; 5, Serovar Hardjo strain Hardjoprajitno; 6, Serovar Muenchen strain Muenchen C90; 7, Serovar Weerasinghe Strain Weerasinghe; 8, Serovar Paidjan strain Paidjan; 8, Serovar Biggis strain Biggs; 9, Serovar Bangkinang strain Bangkinang 1; 10, Serovar Jonsis strain Jones.

## Supplementary Material

Additional File 1 Characteristics of *Leptospira interrogans *reference strains and serovar australis strains and allelic profiles for all strains tested. Click here for file

Additional File 2 *Leptospira interrogans *serovar Australis: clustering analysis of MLVA data part one. Click here for file

Additional File 3 *Leptospira interrogans *serovar Australis: clustering analysis of MLVA data part two. Click here for file
